# Decisional needs for older adults, home health care nurses and dental hygienists during team-based oral health assessments in ordinary home settings – a qualitative study

**DOI:** 10.1186/s12877-024-05367-6

**Published:** 2024-09-23

**Authors:** Jessica Persson Kylén, Sara Björns, Catharina Hägglin, Ingela Grönbeck-Lindén, Laurence Piper, Inger Wårdh

**Affiliations:** 1 Department of Health Sciences, University West, Trollhättan, 461 86 Sweden; 2 Centre for Gerodontology, Public Dental Service, Region Västra Götaland, Gothenburg, 402 33 Sweden; 3 Department of Cariology, Institute of Odontology, Sahlgrenska Academy, University of Gothenburg, Gothenburg, 405 30 Sweden; 4 Department of Behavioural and Community Dentistry, Institute of Odontology, Sahlgrenska Academy, University of Gothenburg, Gothenburg, 405 30 Sweden; 5 School of Business, Economics and IT, University West, Trollhättan, 461 86 Sweden; 6 Economic and Management Sciences, University of Western Cape, Bellville, 7535 South Africa; 7 Department of Dental Medicine, Karolinska Institute, Huddinge, 141 04 Sweden; 8 Academic Centre for Geriatric Dentistry, Stockholm, 112 19 Sweden; 9 Department of Health Sciences, Karlstad University, Karlstad, 651 88 Sweden

**Keywords:** Ageing, Dental care, Expansive learning, Health planning, Oral health, Shared decision-making, Work-integrated learning

## Abstract

**Background:**

Participation by all actors involved in health planning is a prerequisite for person-centred care and healthy ageing. Understanding the multiple knowledge needs and the values that shape oral health assessments in home settings is important both to enable participation in oral health planning and to contribute to healthy ageing.

**Objective:**

The aim of this study was to investigate decisional needs during oral health assessments in ordinary home settings from the perspectives of older adults, home health care nurses and dental hygienists.

**Methods:**

Data was collected in ordinary home settings through 24 team-based oral assessments and 39 brief, semi-structured interviews including older adults (*n* = 24), home health care nurses (*n* = 8) and dental hygienists (*n* = 7). Data was analysed using content analysis with a deductive approach. The analysis was guided by the Ottawa Decision Support Guide.

**Results:**

The analysis revealed that all participants considered participation in decision-making important but until now, older adults might not have participated in making decisions regarding oral health issues. The older adults considered participation important because the decisions had a strong impact on their lives, affecting their health. The professionals considered decision-making important for knowing what step to take next and to be able to follow up and evaluate previous goals and treatments. Organizational and personal barriers for shared decision-making among home health care nurses and dental hygienists were identified. Of the 24 older adults, 20 had different oral health conditions that objectively indicated the need for treatment. An initial important decision concerned whether the older adult wanted to make an appointment for dental care, and if so, how. Another decisional conflict concerned whether and how assisted oral care should be carried out.

**Conclusion:**

It is important for key participants in ordinary home settings to participate in interprofessional teams in home health care. To further anchor this in theory, conceptual models for professionals from different care organizations (municipal care, dental care) need to be developed that also involve older adults as participants. Future research could bridge theory and practice by including theories of learning while exploring interorganizational oral health planning in home settings.

**Supplementary Information:**

The online version contains supplementary material available at 10.1186/s12877-024-05367-6.

## Introduction

Health care systems all over the world face increasing challenges due to the demographic trends towards an ageing population with multiphase needs of care [1]. To enable healthy ageing, professionals across organizations and from separate knowledge domains are expected to collaborate in new ways. Healthy ageing requires exchange of knowledge and collaborative learning among both professionals and patients who are older adults. The growing number of older adults receiving care at home [2, 3] makes interorganizational health planning for this population critical for future health care systems and for person-centred care [4].

Person-centredness supports people’s equal value and stresses that every person is unique [5]. Guidelines in health care and dental care hold that care needs to be further developed towards person-centredness [6–9]. However, when different professions work together there is rarely just one treatment option, especially when considering each patient’s values and preferences. Most people want to participate in decision-making regarding their own health [10, 11]. Shared decision-making is described as a process by which a choice is made by the patient, significant others, or both, together with one or more health care professionals [12, 13]. Shared decision-making has been labelled as the main element in person-centred care [14]. Key elements for shared decision-making are [13, 15]:

- Encourages learning about the patient and offers preference-based treatment options.
- Encourages awareness of health care professionals’ knowledge/recommendations.
- Encourages making the decision.
- Encourages tailoring information.
- Explicitly encourages the use of shared decision-making.
- Defines/explains the problem.
- Creates choice awareness.
- Encourages deliberation (negotiation).

Through shared decision-making, individuals can make a quality decision based on the best available evidence about the risks and benefits of alternatives and what matters most to them [16].

Oral health is crucial for quality of life and wellbeing [17, 18], but is reported to be the most neglected area in nursing research concerning long-term care [19]. Poor oral health is a severe problem among older adults in ordinary home settings [20] and can lead to malnutrition [21], affect the development of diabetes [22], and cause aspiration pneumonia [23] and cardiovascular diseases [24]. Health care professionals have indicated various barriers to oral health work within health care, such as knowledge gaps, negative attitudes, and poorly defined roles and responsibilities [8, 25–29]. Therefore, models are called for to integrate dental care further into the health care domain [8]. When developing models for integrating dental and health care within home health care, a needs assessment could be recommended as a first step [30]. A needs assessment identifies (i) what a patient population needs to make better decisions and (ii) what a population of health professionals needs to improve in the support provided to patients in the decision-making process. Studies that investigate decisional needs in regular home health care regarding oral health issues seem to be lacking.

### Introduction to current practice

#### The Swedish dental care remuneration programme

For people with extensive need of overall care, a dental care remuneration programme is offered in Sweden, regulated by law [31]. The goal of the programme is to support oral health, quality of life and proper nutrition. Through the programme people are offered subsidized dental care and one yearly oral assessment in their homes. In 2022, 12 434 people received an oral assessment through the programme in the west of Sweden [32]. The oral assessments, which are regulated through a collaborative agreement between the Region of Västra Götaland and municipalities, should be carried out with both dental and municipal care staff present. In these assessments, an oral health plan must be outlined and documented on an ‘oral care card’. The ‘oral care card’ is a paper-based protocol that describes the oral status of the person in the programme, including recommendations for everyday oral care. If the person needs dental care treatment, it is also documented on the card. However, recommendations documented on the cards are rarely integrated into the medical records of care in ordinary home settings [33, 34]. This dilemma indicates that there is a good basis for change and learning [35]. Further, there are, to our knowledge, no studies that have investigated these learning aspects, specifically decisional needs during interorganizational oral health planning in ordinary home settings within the framework of dental care remuneration programmes. Elucidating the different knowledge needs and the values that shape oral health planning in home settings can be decisive in contributing to healthy ageing. It can also offer insights into aspects of lifelong learning in working life for health professionals and into the workings of shared decision-making. Thus, the aim of this study was to investigate decisional needs during oral health assessments in ordinary home settings from the perspectives of older adults, home health care nurses and dental hygienists.

## Theory

### The Ottawa decision support framework

In research there exist several frameworks, conceptual models and theories regarding shared decision-making [12]. The Ottawa Decision Support Framework is well-cited [36] and specifically includes the context of the persons facing the decision as well as other people who influence the decision. The framework is theoretically grounded in expectancy value, social support, cognitive and psychological theories [37–44]. Within the framework, a person makes a decision based on knowledge of alternatives, expectations, values, beliefs about the alternatives, and knowledge of decisional conflicts, and is given support and resources to make and implement the decision [36]. The decision process is considered iterative. Different options can further be understood through tailored information as a result of learning from each other during decision-making, where shared values and preferences are emphasized and systematically included in the process [13, 15]. Contradictions, such as reflecting on different alternatives in oral health assessments, can manifest as dilemmas in workplaces. The social dilemmas highlighted in this article have been well documented in research. Evidence suggests a deficiency in knowledge of oral health and oral care and that attitudes, roles and responsibilities regarding oral health in relation to older adults require enhancement [25–29, 45].

This approach provides a good basis for expansive learning, where participants can be involved in the co-creation and development of a radically new, broader and more complex knowledge of their activity [46]. When emphasizing a socio-cultural approach to interorganizational health planning in home care, it is important to take into account the environment – as the framework suggests [36]. Different participants shape and are shaped by each other by learning and sharing objects (e.g. what creates meaning for each participant). Through the Ottawa Decision Support Framework, a five-step guide for decision-making in clinical practice has been provided. Bridging theory and practice, the five steps will contribute by guiding a deductive analysis in the results section, which will extract new knowledge about decisional needs for oral health planning in home settings.

## Methods

The study design is qualitative with a deductive approach. The data was collected through 24 interprofessional, team-based oral assessments performed in ordinary home settings of older adults [47, 48]. In addition, transcriptions of 39 brief interviews also served as data.

### Participants

The teams consisted of (i) one older adult, (ii) one home health care nurse (responsible for the home health care of the older adult) and (iii) one dental hygienist from the dental care remuneration programme (Table 1). The data was collected in October and November 2022 in seven municipalities in western Sweden. Both rural and urban municipalities were included. Table 1 shows the number of inhabitants in each municipality [49] and empirical data for the participants.

**Table 1:** Number of residents in each municipality and empirical data on the participants

	Munici-pality A	Munici-pality B	Munici-pality C	Munici-pality D	Munici-pality E	Munici-pality F	Munici-pality G	Total
Population	42 199	114 445	33 252	5 646	49 068	39 852	59 274	n/a
Home health care nurses (n)	1	2*	1	1	1	1	1	8
Work experience (years)	10	17, 10	18	20	8	15	2	12.5 (mean)
Dental hygienists (n)	1	1	1	1	1	1	1	7
Work experience (years)	30	33	18	38	11	6	32	24 (mean)
Older adults	3	3	4	3	4	4	3	24
Age (years)	91, 99, 87	87**, 90**, 92**	91, 81, 91, 63	69, 76, 91	88, 86, 79, 87	91, 91, 71**, 82	81, 81, 79	84 (mean)
Enrolled in home health care (years)	2, 5, 2	2, 2, 2	2, 3, 1, 2	4, 2, 2	7, 5, 2, 3	7, 0, 5, 0	6, 3, 4	3 (mean)
Sex	3 female	3 female	4 female	3 female	3 female, 1 male	2 female, 2 male	2 female, 1 male	20 female, 4 male
Wheelchair (n)	1	1	-	2	2	1	2	9
Participating from bed (n)	-	1	-	1	-	1	-	3
Blind (n)	-	-	1	-	1	-	-	2
Identified as entitled to dental remuneration programme as a result of the study	-	3	3	2	1	-	-	9

* There were two nurses scheduled for the oral assessments; one nurse participated in two assessments, and the other participated in one

** A relative to the older adult was also present during oral assessments and interviews

n/a – not applicable

**Fig. 1:**
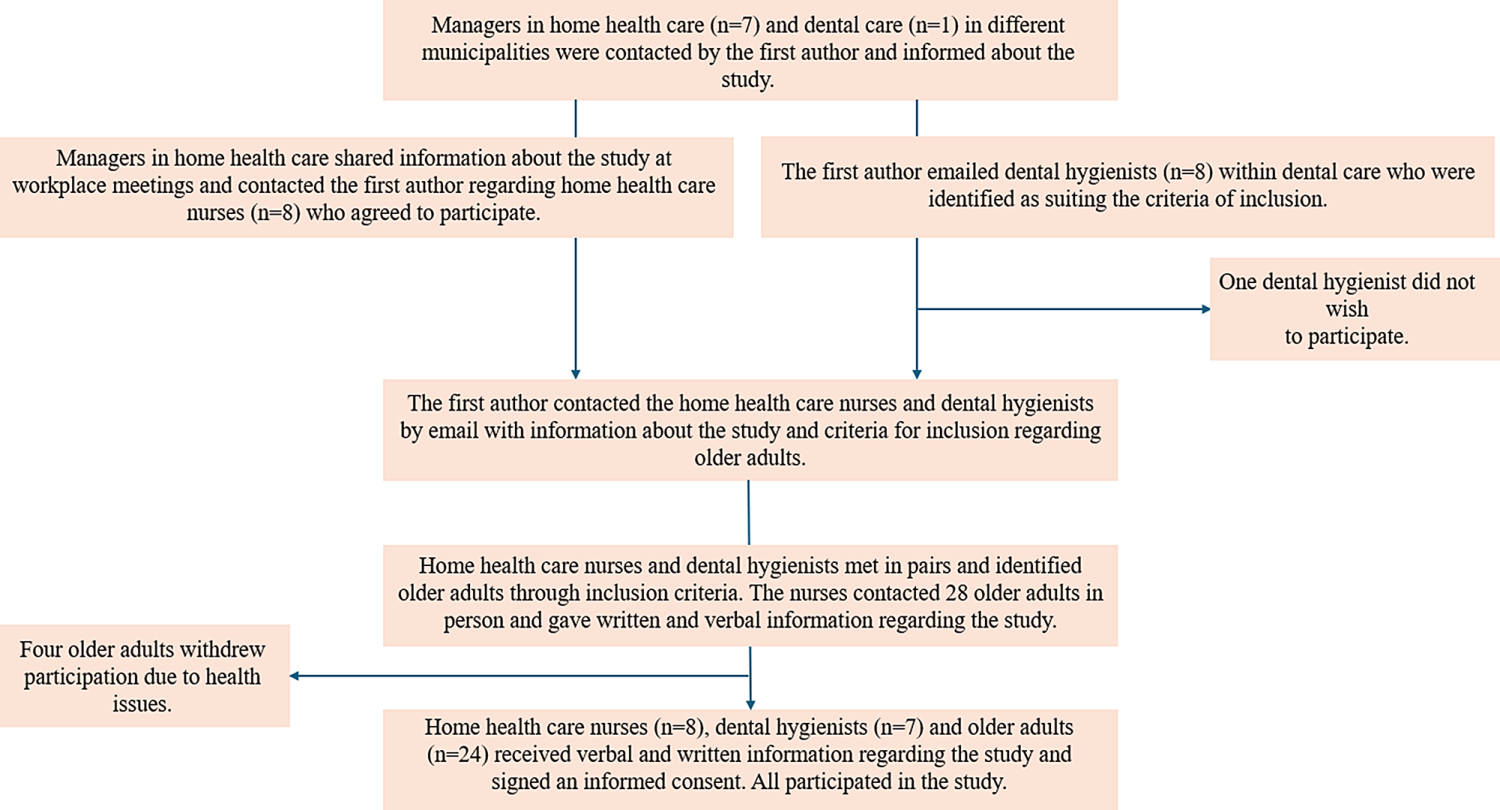
Process of participant recruitment

The process of participant recruitment is visualized in Fig. 1. Seven managers for municipal home health care organizations and the manager of the dental care remuneration programme in the public dental care region Västra Götaland were initially contacted by email regarding the study. They all gave their consent for their nurses and dental hygienists, respectively, to participate in the study if they so wished and they also informed them about the study. Eight nurses were purposefully selected because of their experience of working as home health nurses. All wanted to participate. Eight dental hygienists working in public dental care in the west of Sweden were purposefully selected because of their long experience of working in the dental care remuneration programme. Seven dental hygienists agreed to participate.

The home health care nurses and dental hygienists formed pairs that identified older adults enrolled in home health care. They had not been working together before. The inclusion criteria for the older adults were to be able to (i) speak and understand Swedish, (ii) be enrolled in home health care and (iii) enrolled in or entitled to the dental care remuneration programme, but (iv) not be regarded by the home health care nurses to have cognitive impairment. The home health care nurses contacted the patients in person and asked permission for their participation. Four older adults who had agreed to participate withdrew their participation due to health issues. All participants (older adults, home health care nurses and dental hygienists) received written and oral information and were asked to participate on a specific day for the oral assessments, and all gave their written and oral consent to participate.

### Oral assessments

The oral assessments lasted approximately 25 min and involved the following phases:

I. Self-reported health and oral health of the older adults (‘*How do you feel?*’ and ‘*How do you feel in your mouth?*’ including follow-up questions). This phase was conducted to get the older adults’ views on oral health, establish a trustful alliance [50] and involve them as active partners in the teams.
II. Risk assessment with the Revised Oral Assessment Guide-Jönköping (ROAG-J) [51]. ROAG-J is an oral health assessment instrument primarily for use by non-dental health professionals [52]. The validity and reliability of the ROAG-J have been shown to be good [51, 52]. ROAG-J includes nine items with answer options from 0 to 3. Zero means ‘not applicable’, grade 1 ‘no problem’, grade 2 ‘oral health problem’/‘risk’ and grade 3 ‘severe oral health problems’/‘risk’ (Table 2). In addition to the risk assessment, planned preventive actions are included in ROAG-J.
  For those who on the ROAG-J items ‘teeth’ and ‘gums’ were assessed to have poor oral hygiene and/or gingivitis, the cause of the inability to manage daily oral care was also investigated.

**Table 2:** ROAG-J [51]; the nine items and the grades^1^

Item	Grade 0	Grade 1	Grade 2	Grade 3
VOICE	Not applicable to judge	Normal	Dry, hoarse, smacking	Difficulty speaking
LIPS	-	Smooth, bright red, moist	Dry, cracked, sore corners of the mouth	Ulcerated, bleeding
MUCOUS MEMBRANES	-	Bright red, moist	Red, dry, or areas of discoloration, coating	Wounds with or without bleeding, blisters
TONGUE	-	Pink, moist with papillae	No papillae, red, dry, coating	Ulcers with or without bleeding, blistering
GUMS	No gums, only mucous membranes	Light red and solid	Swollen, reddened	Spontaneous bleeding
TEETH	No natural teeth	Clean, no visible coating or food debris	Coating or food debris locally	Coating or food debris generally, broken teeth
DENTURES	No prosthetics	Clean, functioning	Coating or food debris	Not used or malfunctioning
SALIVA^2^	-	Runs freely	Runs sluggishly	Does not run at all
SWALLOWING	Not applicable to judge	Unimpeded swallowing	Minor swallowing problems	Pronounced swallowing problems

^1^ Grade: 0 = not relevant to assess, 1 = healthy or normal condition, 2 = moderate change or divergence, 3 = severe changes or divergences

^2^ Examined by running a mouth mirror over the inside of the cheek

For this, parts 2 and 3 of the Oral Hygiene Ability Instrument (OHAI) were used [53]. Part 2, is a brief clinical examination to assess dry mouth, oral status, and muscular and spatial oral functions, mainly for exploring the oral clearance aspects of problem with daily oral care. Part 3, is an observation of tooth brushing activity that aims to assess whether it can be e.g. impaired fine motor skills or cognitive function that negatively affects oral hygiene. The following actions were explored:

- Pick up toothpaste.
- Unscrew cap.
- Apply toothpaste.
- Brush/pick teeth properly.
- Pick up interdental cleaning stick/brush.
- Use interdental cleaning stick/brush.
- Rinse mouth with water [53].

Based on this, the home health care nurses and dental hygienists – as part of OHAI – evaluate the extent to which ten possible influencing factors (cognitive function, frailty, motivation, vision, fine motor skills/coordination, knowledge of oral hygiene, spatial ability, oral clearance, emotions, balance problems) are the cause of the participants’ impaired oral hygiene skills in order to be able to suggest appropriate actions [36, 46].

III. Health planning based on phases I and II. Having a shared tool, such as a shared protocol for oral assessment and documentation, during health planning in ordinary home settings is important for both planning and decision-making [54]. The interactions within the teams were initiated through these phases. The phases were also integrated in the shared paper-based prototype for a new ‘oral care card’ on a digital platform. The prototype was developed based on previous studies [34, 55]. No prior educational efforts were conducted on how to implement the phases within the oral assessments and the paper-based prototype, and all phases were performed in collaboration decided within the teams.

### Data collection

The data consists of documentation from 24 oral assessments. The older adults were mainly seated during the oral assessments (ROAG-J, OHAI Part II), however three older adults participated when laying in their beds (Table 1). For the participants with poor oral hygiene, the toothbrushing activity in Part III of the OHAI was performed as usual, that is, normally in the bathroom by a sink. Instruments used at oral assessments were torch and dental mirror. To secure that the process followed the study protocol, two researchers observed all the oral assessments without interacting (JPK, AS). In four of the teams, relatives (three daughters and one wife) participated based on the wishes of the older adults. After each oral assessment the older adults (occasionally together with relatives) were briefly interviewed by JPK and AS in their homes (*n* = 24). The interview guide was:

- *Could you please describe what happened during the oral assessment? How did that feel?*
- *What is important to consider before entering your home as a professional?*
- *What is important when leaving your home as a professional?*
- *Is there anything else that is important for us to know*,* regarding oral health planning?*

The home health care nurses and dental hygienists were individually interviewed (mean length: 14 min) at the home health care nurses’ offices after participating in the oral assessments they were assigned. The interview guide for home health care nurses and dental hygienists was:

- *Could you please describe this workday?*
- *Were there any differences in regard to how you usually work? If so*,* what/how?*
- *Would you like to integrate oral health perspectives further in home health care?*
- *If so*,* how and why?*
- *Do you have any main learnings to share from this workday?*

### Data analysis

Background data was given in frequencies, means and percentages. Interview data was analysed according to qualitative content analysis [56, 57]. A deductive approach was used where the Ottawa Decision Support Framework guide [36] served as the basis for the analytical framework. The five steps in the guide were used as bases when the categories in the analysis were formulated: (i) Recognition that a decision needs to be made; (ii) Understanding information in relation to the decision; (iii) Clarification of values and identification of preferences according to these values; (iv) Consideration of resources, including social influences that affect the decision; and (v) Formation of an action plan. Consequently, five research questions were formulated when analysing the data:

1. How did the participants recognize that a decision needs to be made?
2. How did the participants understand information in relation to the decision?
3. How did the participants clarify values and identify preferences according to these values?
4. How did the participants consider resources, where social influences that affect the decision also were included?
5. How did the participants form an action plan?

All transcriptions were read repeatedly and independently by JPK and SB, and a first analysis was performed and then discussed together. Thereafter, it was iteratively discussed several times by all authors. All authors agreed on the final analysis. The ROAG-J assessments were analysed and presented in frequency and percentage based on identified risk (grade 2 and 3) in relation to ROAG-J items.

## Results

### Background information

Of the 24 older adults, 20 (83%) were assessed to have at least one oral health problem according to ROAG-J and of these, seven (35%) had at least one severe problem (grade 3) (Table 3). In supplementary material suggested actions from professionals after the ROAG-J and oral hygiene assessments are visualised.

The most common oral health problem was for ‘Teeth’ followed by the item ‘Saliva’ (dry mouth). For the item ‘Dentures’, no oral health problems were registered.

Seven of the 24 older adults had poor oral hygiene and/or gingivitis according to ROAG-J (‘Teeth’ and ‘Gums’, Table 2) and were therefore assessed with the OHAI. The causes for having poor oral hygiene (more than one cause was possible per person) were concluded by the professionals to be poor oral clearance (*n* = 4), frailty (*n* = 3), fear of pain and/or bleeding (emotions) during oral hygiene activity (*n* = 2), lack of motivation (*n* = 1), impaired fine motor skills (*n* = 1), poor balance (*n* = 1), impaired cognitive function (*n* = 1) and lack of knowledge about oral hygiene (*n* = 1). In addition, the OHAI assessment showed that the environment in the bathroom for three of the older adults was not adapted to their needs and could therefore in various ways make the oral hygiene more difficult.

**Table 3:**
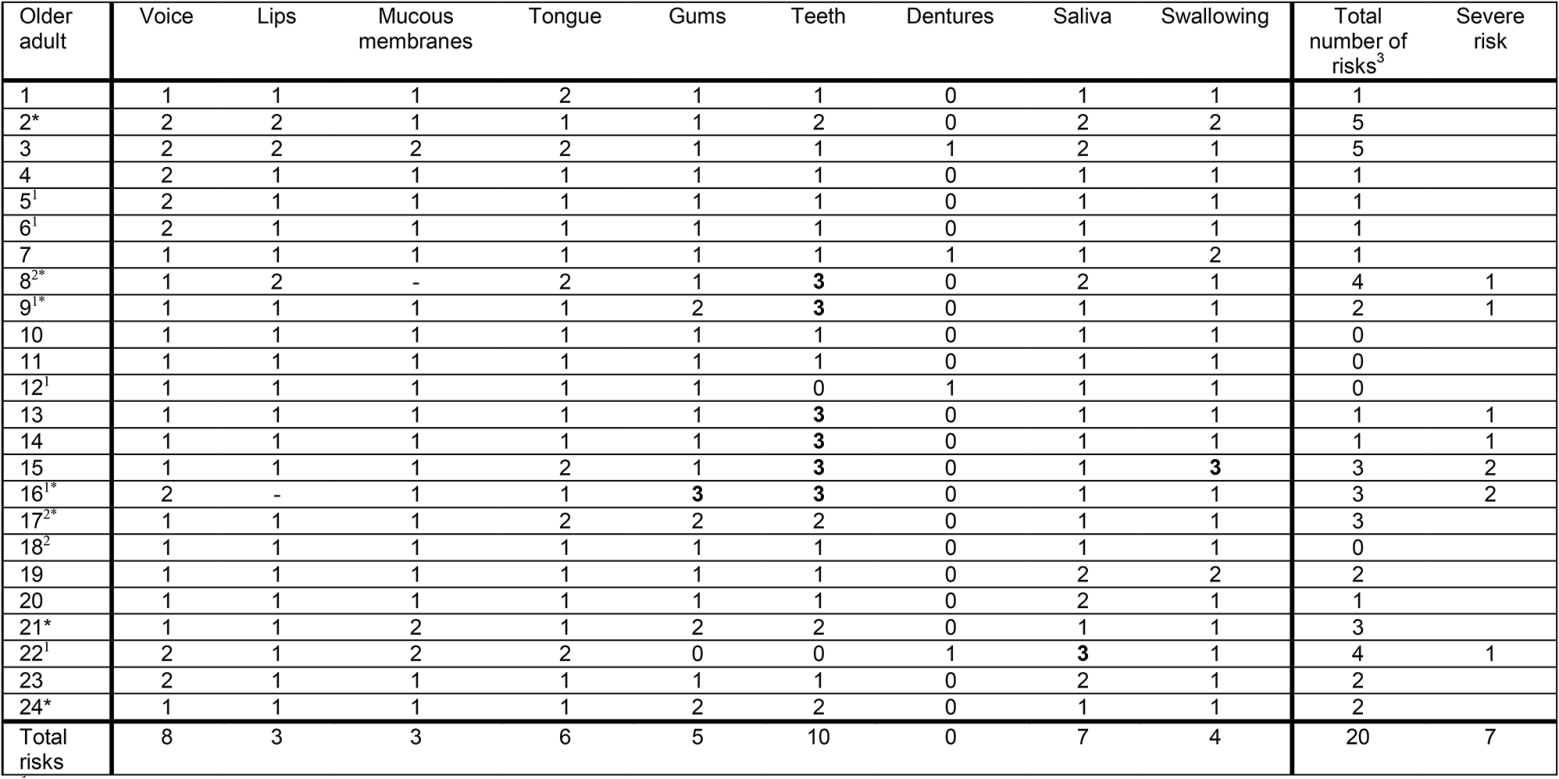
Outcomes from the ROAG-J assessments according to the nine items in the instrument, including whether there was an established dental health care contact

^1^No regular dental health care contact

^2^Postponed dental health care appointments

^3^Number of oral health problems (ROAG-J grades 2 and 3 = risk)

* Poor oral hygiene (but some older adults scored 3 on item “Teeth” due to fractured teeth)

Many older adults reported good oral health. However, some experienced severe problems. In these cases, the suspicion of the presence of oral diseases seemed to frighten the older adults, and this affected their daily lives in crucial ways, such as being afraid to chew.

*Patient 14 Team D (87 years): It* [the bridge in the upper jaw] *just came loose. // But the one*,* on the other side here I feel that it is a little rough underneath. So I am really scared. I barely dare to bite into anything because it* [the bridge in the upper jaw] *just came loose! // And all teeth have broken within a few months*,* half a year.*

The two main decisions that the teams faced during the oral health assessments were: (i) whether the older adults wanted to visit dental care clinics, mainly due to existing oral conditions (Table 2), and/or (ii) whether they wanted assistance in various aspects of oral care.

### Recognition that a decision needs to be made

The older adults were unaware that they could participate in decisions concerning their oral health; neither relatives nor professionals had realized that persons other than dental professionals could participate in decision-making regarding oral health issues:

*Interviewer: You are going to get a lot of work done on your teeth*,* too*,* as I understand it?*

*Patient 15*,* Team E (87 years): Yes*,* I am going to get teeth*,* right? They* [dental care personnel] *are going to put in teeth where they are missing?*

*Daughter: We’ll have to see what they* [dental care personnel] *decide once they have made the decision.*

Decision-making was considered as something the older adults or relatives were informed about, not involved in. However, the older adults expressed that they wanted to participate in decision-making and discuss different treatment options.

The home health care nurses seemed to be unsure about how to handle decisions regarding oral diseases. One home health care nurse had contacted a dental clinic regarding an older adult, but since the older adult could not be transported to a dental clinic, she got the impression that dental care organizations did not treat patients with special care needs.

Concerns regarding who was responsible for decisions about oral health were discussed by both the home health care nurses and the dental hygienists. One barrier identified by home health care nurses was that they felt overloaded with responsibilities. As such, oral health became more peripheral:

*Nurse*,* Team C: At times you can feel a little like a naggy nurse*,* you know. Who kind of says that you would need this*,* or this would be good for you … but*, yeah. *sighs* Then you don’t get that far … Because you’re kind of responsible for so many things and *sighs* This thing with the teeth,* well*,* unfortunately it ends up a bit further down* [on the list of priorities].

The home health care nurses and dental hygienists agreed that health planning was important because it determined what steps to take next and helped to understand what needed to be followed up. Nevertheless, several aspects regarding decision-making across organizations were described as complicated by the professionals, for example confidentiality and documentation.

### Understanding information related to the decision

The professionals stated that the team-based oral assessments taught them to consider and understand new aspects of care, and tailored information emerged as a result of this.

*Nurse*,* Team D: It was really interesting and a learning experience to be working together with the dental hygienist. She could teach me. And I think that maybe we could teach each other in some way … // … No but with the oral assessment*,* I could see how she does it in a completely different way than trying to find out myself.*

Each organization (i.e. municipal health care and public dental care), with its different rules and regulations, became more visible through the spontaneous dialogues during the collaboration. The oral assessment process became an opportunity to reflect together, understand tailored information and ask questions.

The records in home health care and dental care are not synchronized, and this was seen as jeopardizing the safety of older adults enrolled in home health care. The fragmented system of home health and dental care constituted a major challenge when it came to discussing treatment options, which sometimes could lead to tensions between the different professions. There was also a lack of information about which dental clinic the older adults were registered at, and where to call if oral problems arose.

*Nurse 1*,* Team B: Who are we supposed to contact if it looks abnormal*,* just a thing like that. As it is today*,* I know that staff can call* [the dental clinic], *kind of. But at the same time*,* we are told elsewhere that the patient*,* or the staff doesn’t get anything back from* [dental care]. *What have we assessed*,* what have we looked at*,* well … And not me as a nurse either*,* I never get any feedback*,* even though I perhaps have assessed according to ROAG that this needs to be done*,* I won’t get any feedback either.*

Some participants suggested that the care processes were in need of further integration that could be supported by a shared digital platform, integrated in existing systems for documentation, to enable better access to information related to medical decisions.

### Clarification of values and identification of preferences according to these values

It was important for the teams to be able to describe the role of oral health in ordinary home settings. The dental hygienists described that they had very limited knowledge about the older adults. Sometimes limited understanding could lead dental hygienists to make assumptions regarding older adults’ abilities or inabilities.

Being able to evaluate oral hygiene ability in a structured way in a home setting was considered important when it came to understanding older adults’ total situation. Regarding the personal aspects of oral hygiene, many older adults expressed that they always performed oral care themselves. The oral cavity was considered to be of high integrity.

*Patient 16*,* Team B (90 years): Is some 20-year-old supposed to help me brush my teeth?!?*

*Interviewer: Yes*,* how do you feel about that?*

*Patient: No*, I’m not really sure whether they would be so good at it. *laughter*.

Many older adults discussed how trust and continuity were important to them when it came to oral health. None of the older adults asked for assistance with ‘hands-on’ oral care. However, they asked for different care options, such as help with cognitive support, for example remembering to brush or get help booking appointments at dental clinics. They also asked for help in getting fluoride in a smaller, lighter bottle.

### Consideration of resources, including social influences, that affect the decision

The older adults described how they wanted the care to be provided. For example, before entering their home, many older adults emphasized the importance of proper introductions that included who everyone was, what they planned to do, and why.

*Patient 20*,* Team C (91 years): Yes*,* then I’ll want them to call first. And inform me about the purpose of the visit. And whether they want anyone else to be there. Or if it’s enough with just me. // To just show up*,* which some people do just like that. I don’t like that.*

The older adults wished for written information before the oral assessments, but some preferred getting the information through a phone call.

All participants stated that it was important to document what was agreed upon. Some older adults wanted relatives to also get the information, others did not. The participants were generally concerned about the most important outcomes of the assessment in regard to what was valuable to them. The older adults often requested that the information would be given in few words and a large font. The professionals wanted the information to be integrated into their digital systems.

The professionals in the teams were used to working mostly on their own. There seemed to be no obvious method or system to make decisions, understand and learn from each other.

*Dental hygienist*,* Team F: The biggest difference was that we so to speak were two professions. Otherwise*,* as a dental hygienist I think you’re quite independent and work on your own a lot. So that was a big difference today*,* that we kind of were two who were to take care of this together. And that has a lot of advantages. But we’re not used to it*,* either.*

Conducting oral assessments in teams seemed to support the sharing of the assessments’ results and seemed to influence the decision-making process in such a way that the professionals did not feel alone in making decisions. The team approach was a major change in how home health care nurses experienced collaboration with dental health care.

The nurses considered oral hygiene as a task outside of health care, so issues affecting oral health could be lost in a grey zone between health care and dental care. The understanding of what was most important to each person regarding oral health issues was sometimes regarded as being part of home health care and sometimes not.

### Formation of an action plan

It was a new experience to perform oral assessments in collaboration, guiding each other and using instruments which both professional groups could understand and follow.

*Nurse 4*,* Team E: I think that it was an eye-opener to be working together with a dental hygienist as a nurse. We looked at the same things quite a lot. We’re the same. But still*,* to get to listen to her assessment and see what she was thinking. That was really useful. Even though we made the same assessment on ROAG*,* many of the other things were still useful to know. That I hadn’t thought of.*

A request was made for oral assessments to be coordinated in a simple way so that the action plans could be followed up and revised in an interprofessional and interorganizational process. The creation of an action plan was perceived as a very complex task, involving many aspects of information and coordination, such as oral health status, general status, coordination of care visits, recommended products and follow-ups. When there was no common ground in daily activities, no shared decision-making could take place either. The professionals also stated that different legislations created barriers, where oral health in home health care was placed in a grey zone.

Older adults, home health care nurses and dental hygienists all expressed a desire for innovative options, such as mobile dental health care teams performing dental care in home settings, or digital consultants integrated in a future shared digital tool that enabled the sharing of an action plan, easy to document and schedule. Dental hygienists were requested as team partners for future home health care.

None of the older adults stated that they did not want to participate in decision-making, nor did they not want information about themselves. On the contrary, many expressed a wish to participate, and to receive tailored information.

*Patient 5*,* Team G (81 years): I think that almost the most important thing is that they* [the staff] *think about me a little. How I am feeling.*

## Discussion

The results revealed that all participants considered participation in decision-making to be important for both improved health care and participant wellbeing. However, the older adults were not used to being invited to participate in decision-making regarding oral health issues. Interprofessional, team-based oral assessments seemed to open up learning opportunities triggered by the diverse knowledge, values and preferences of the participants in the oral assessment process. The results also highlighted that decision-making could enable learning, with outcomes as tailored information for all participants according to their need. The results will be discussed through decisional needs assessments’ dual purposes, namely (i) what a patient population needs to make better decisions and (ii) what a population of health practitioners needs to improve in the support they provide to patients during decision-making [30].

### What a patient population needs to make better decisions

Older adults and actors involved in their daily life need to be included in decision-making during oral assessments in ordinary home settings to improve health care. By systematically involving older adults in decision-making processes as much as the older adult wish or is possible to do, a sense of reciprocal learning, autonomy and empowerment can be considered to be integrated into oral assessments. This enhanced autonomy and empowerment is an addition to the often improved medical outcomes. To accomplish this, future models for oral assessments need to recognize that home settings are complex arenas where many stakeholders are included in care processes. In previous research it was concluded that it was crucial to integrate the older adults’ preferences and values regarding their oral health in oral assessments [34]. Inspired by OHAI [53] to assess ability during the oral hygiene task in home settings, new opportunities for learning were opened. For example, the results show that older adults were recommended to use a chair in the bathroom when balance was poor. As such, the results indicate that professionals learn from older adults by including them and their surroundings in decision-making. This needs to be further explored and integrated in a structured and methodological way in future research in order to enable person-centred care.

Because of the diversity of older adults in the study, with many having poor oral health but differing preferences regarding whether and how to address this, the importance of bridging practice with theories based on person-centred care is emphasized [58]. Another critical aspect is to co-create working models with multiple key stakeholders to further bridge challenges in practice and theory. For example, including the lived experience of being an older adult in future research could be regarded as both important and ethically fundamental [59]. This study shows that older adults with home health care want to be a part of the process of oral assessments; they want to know who is coming, when, and why. They also want information about what was said. This could be considered as very basic aspects of care, but in a complex care setting, such as interorganizational oral assessments, this might be complicated to achieve. Also, future research involving older adults with impaired cognitive functions are crucial to conduct, for not excluding important populations from evidence-based research. Health plans for oral assessments in home settings are probably perhaps not yet introduced. It is also important to further include and study the learning aspect of interorganizational work [46].

### What a population of health practitioners needs to improve in the support they provide to patients during decision-making

In line with the existing body of knowledge, it was found that oral health care outside of dental care clinics seems complicated [7, 8, 25–29, 34]. Responsibilities, knowledge, financing, attitudes and regulations have all previously been identified as barriers for collaboration between dental health care and other health care professions. This gap between organizational expectations and practical realities might be due to a lack of specific training, unclear guidelines, or the overwhelming nature of other responsibilities that take precedence in the workload. In such scenarios, the prioritization of tasks becomes a significant challenge, and areas like oral health can be overlooked unless they are explicitly integrated into care plans and protocols.

This article contributes to theory by emphasizing the challenges single health care professionals face in collaboration when there is no common platform and base for sharing values and preferences [34]. Theory-based conceptual models for interprofessional shared decision-making do exist, although they are mainly focused on professionals within the same organization [60]. However, the results indicate that models for integrating dental care professionals into home health care, and bridging different organizations with different knowledge domains, also need to acknowledge the additional complexity of not sharing an everyday common ground, for example the same medical records. Nor do these professionals share a common educational background, management, regulations, or arena for developing shared understandings in everyday work. For the increasingly ageing population that has many preserved natural teeth but often loses regular dental contact [61], there may be a great need to develop conceptual models further. A practical implication of this study is to further develop models for integrated oral care in home settings, which also are anchored in learning theories and conceptual models for interprofessional shared decision-making.

In working life, policies for home health care and dental care emphasize lifelong learning. This study supports the view that decision-making in many ways is based on learning about and from each other, discussing the pros and cons of treatment and also acknowledging each other’s expertise. When integrating a common instrument such as ROAG-J for professionals to guide each other, a shared understanding and language seem to enable new, sometimes unexpected, learning opportunities, almost as tacit knowledge when working side by side. This is in line with expansive learning theory [41], where collaboration can lead to actions that could not have been planned for on one’s own.

### Strengths and limitations

The data collected here is both the strength and the limitation of the study. Including 39 interviews, 24 oral assessments and 24 ROAG-J, it could be considered complex and large. The data has assisted in visualizing decision-making in home settings in various ways. One strength is the inclusion of older adults, describing their point of view regarding oral health in their own home. This may have influenced them to feel comfortable in participating, as the home as an arena is described as an empowering environment for older adults [62]. One limitation was the way the inclusion of home health nurses and dental hygienists was done: they all explicitly wished to participate, which surely affected the results. Not every professional might be as happy as they were to explore and learn in new team settings. However, creating knowledge by testing and exploring a new, not all-set model, participants eager to co-develop and participate had its advantages as well.

## Conclusion

Participation in oral health planning in home settings involving key stakeholders is important for further achieving person-centred care. Older adults want to participate in decision-making regarding their oral health. A prerequisite for this seems to be to enter oral assessments with an expansive learning approach. Older adults, home health care nurses and dental hygienists can expand their learning and competencies by working side by side in home settings, embracing the activities within the home setting of the older adults. To further support this through theory, conceptual models for professionals from different care organizations, such as municipal and dental care, need to be developed. Using team-based, interprofessional oral assessments as a foundation for gaining knowledge, and also understanding the values and preferences of other participants, can be considered an area of development for enabling person-centred care and healthy ageing.

## Electronic supplementary material

Below is the link to the electronic supplementary material.

Supplementary Material 1

## Data Availability

The datasets used and/or analyzed during the current study are available from the corresponding author on reasonable request.
