# Supplementary material for: Decisional needs for older adults, home health care nurses and dental hygienists during team-based oral health assessments in ordinary home settings – a qualitative study
Source: BMC Geriatr. 2024 Sep 23;24:779. doi: 10.1186/s12877-024-05367-6 (PMC11421118; doi:10.1186/s12877-024-05367-6)
Supplement: Supplementary file 1 — Supplementary Material 1 [file 12877_2024_5367_MOESM1_ESM.docx]

Supplementary materials

**Table 3.** Suggested actions from professionals after the ROAG-J and oral hygiene assessments.

| **Suggested action** | **Number** |
| --- | --- |
| Make new appointment at a dental care clinic | 6 |
| High fluoride product (rinse or paste) | 5 |
| Stimulation products for saliva | 5 |
| Substitute products for saliva | 4 |
| Make appointment with general physician | 3 |
| Toothpaste for treatment of aphtous stomatitis | 1 |
| New toothbrush | 1 |
| Assistance with oral care | 3 |
| Assistance with applying toothpaste | 1 |
| Reminder to perform oral hygiene | 1 |
| Chair in bathroom for not falling during oral hygiene | 1 |
